# Cross-kingdom dialogs in the gut: Integrating bacterial pathogens, helminths, and microbiota interactions for immune homeostasis

**DOI:** 10.1371/journal.ppat.1013494

**Published:** 2025-09-11

**Authors:** Suhui Hu, Zhenzhen Liu, Wenchao Yan, Rongxian Guo

**Affiliations:** College of Animal Science and Technology, Henan University of Science and Technology, Luoyang, China; University of Utah, UNITED STATES OF AMERICA

## Abstract

The interactions between bacterial pathogens, helminths, and commensal microbiota in the gut form a complex ecological network that profoundly impacts host immunity and health. Pathogens employ strategies such as type VI secretion systems (T6SS) and inflammation induction to evade colonization resistance, disrupt microbial balance, and establish self-benefit ecological niches. These interactions involve competition with commensal bacteria and helminths, which play a critical role in maintaining gut homeostasis by occupying ecological niches, competing for nutrient, and supporting the mucus barrier. Meanwhile, helminths can modulate commensal bacterial gene expression, metabolic activity, and survival by secreting excretory–secretory products. In addition, by inducing a Th2 immune response, helminths can enhance the intestinal mucosal barrier, alter the gut microbiota composition, and thereby inhibit bacterial pathogen colonization. Interestingly, helminths and pathogens can exhibit synergistic or competitive relationships. For instance, *Ascaris lumbricoides* may provide a survival niche for *Vibrio cholerae*, while helminths can also indirectly inhibit pathogenic bacteria through immune modulation. These intricate interactions influence gut microbial composition, digestion, and immune function, and are closely associated with diseases. Future research should focus on elucidating the molecular mechanisms underlying these interactions. Understanding the interactions between pathogens, helminths, and commensal microbiota not only provides novel insights into maintaining host immune homeostasis but also establishes a theoretical foundation for future development of gut health intervention strategies.

## 1. Introduction

The intestinal tract is a complex ecological environment where host and microbial inhabitants have coevolved over millennia, leading to mutually beneficial or competitive interactions [1]. Research reports have indicated that the gut microbiota plays a role in supporting nutrient acquisition and metabolism, pathogen defense, as well as shaping the development and responses of immune cells [2].

Bacterial pathogen infections significantly disrupt the balances and functions of intestinal commensal microbiota through various mechanisms. For example, pathogens impair the growth of commensal bacteria by competing for essential nutrients such as short-chain fatty acids (SCFAs) and occupying ecological niches [3,4]. Pathogens can also eliminate commensal bacteria through the type VI secretion system (T6SS) or modify the intestinal environment by inducing host inflammation, thereby creating conditions favorable to themselves but detrimental to commensal bacterium [5]. Moreover, bacterial pathogens degrade intestinal barriers and interfere with the metabolic activities of commensal bacteria, weakening their protective roles in the host. These combined mechanisms disrupt intestinal homeostasis, potentially leading to microbiota-related diseases [6,7].

Furthermore, studies have confirmed that specific microbiota are essential for the hatching of *Trichuris muris* eggs and enhance the infection of *Heligmosomoides polygyrus* in mice [8,9]. Helminth infections can alter the composition and abundance of gut bacteria in their hosts. For instance, the excretory–secretory (ES) products of *Ascaris suum* and *H. polygyrus* exhibit diverse antibacterial activities, such as bacterial growth inhibition, biofilm disruption, and agglutination [10,11]. Additionally, Ascaris has evolved counter-regulatory mechanisms that prevent excessive inflammatory responses caused by bacterial translocation and tissue damage during larval migration [12]. Interestingly, helminths and pathogenic bacteria may also have symbiotic relationships. For example, the free-living *Caenorhabditis elegans* can derive nutrition from microbial components [13]. Moreover, *Ascaris lumbricoides* has been colonized by *V. cholerae* isolated from cholera patients, suggesting that the nematode intestine could act as a survival niche for bacterial pathogens [12,14]. These interactions between pathogenic bacteria and helminths contribute to the creation of an optimal ecological niche for survival.

The intestinal tissue consists primarily of immune cells, epithelial cells, and stromal cells, which together maintain the gut barrier, preventing pathogen invasion and stabilizing the intestinal environment [15–17]. Immune defense against microbial pathogens is referred to as type 1 immunity, which primarily relies on the direct killing of pathogens or infected host cells. The adaptive component of this immunity is mediated by type 1 and th17 cells, cytotoxic T cells, as well as IgM, IgA, and several IgG antibody classes. This type of immunity is characterized by elevated levels of the cytokines IL-12, IL-22, IL-23, IFN-γ, and IL-17. [18]. In contrast, helminths primarily mediate type 2 immunity for defense, relying on barrier defenses and IgG1 antibodies, along with multiple components of the innate immune system, including epithelial barriers, innate lymphoid cells (ILCs), eosinophils, mast cells, basophils, and alternatively activated macrophages (AAMs) [18,19]. To ensure long-term coexistence with their hosts, helminths modulate inflammatory responses by inducing regulatory T cells, AAMs, and releasing anti-inflammatory cytokines such as IL-10 and TGF-β [20–22]. Interestingly, intestinal stromal cells interact with various epithelial cell populations through the basement membrane, playing a crucial role in supporting epithelial cell proliferation and functional maintenance [17]. This regulatory process is primarily mediated by soluble mediators, including specific communication between stromal cells and epithelial stem cells [23]. Recent research data demonstrate that *Wnt* signaling derived from stromal cells plays a dominant role in maintaining epithelial stem cell homeostasis [24]. Notably, stromal cell support for intestinal epithelial differentiation and function is not merely a stationary, passive process. Sophisticated mouse colitis models have revealed that during inflammatory responses, stromal cells actively redistribute to the peri-cryptal region and initiate epithelial barrier repair through prostaglandin E2 secretion [25].

This review primarily explores the intricate interactions in the intestine, focusing on the mutualistic and competitive relationships between intestinal commensal microbiota, bacterial pathogens, and helminths. It highlights the mechanisms by which intestinal pathogens disrupt the balance and functions of commensal microbiota, as well as the interactions between helminths and commensal bacteria, including how helminths regulate commensal microbiota composition and influence host immune response. This review analyzes the interactions between bacterial pathogens, helminths, and commensal bacteria in the gut immune ecosystem. It offers insights for studying gut balance and developing treatments for related diseases.

## 2. The intestinal ecosystem: A stable niche for microbial crosstalk

The intestine is a complex organ consisting of specialized epithelium, nerves, immune cells, blood, lymphatic vessels, smooth muscle, and provides nutrients and a hospitable niche for the trillions of microorganisms. The gastrointestinal tract comprises two main regions: the small and large intestines, with the innermost mucosal layer playing a pivotal role. This mucosa, made of a single cohesive layer of columnar epithelial cells, is responsible for secreting mucus and absorbing nutrients [26]. Embedded within this layer is the lamina propria, which contains blood vessels, lymphatics, nerves, and lymphoid tissue, as well as numerous intestinal glands [1]. These glands secrete intestinal fluids that aid in digestion and nutrient absorption. Supporting the mucosal layer is the submucosa, composed of loose connective tissue enriched with blood vessels, lymphatics, and nerve fibers, crucial for maintaining the digestive, absorptive, motility, and sensory functions of the intestine. The outermost layer, the muscularis externa, is divided into inner circular and outer longitudinal muscle layers, facilitating intestinal peristalsis and food propulsion.

In the intricate and sophisticated microecological system of the intestine, different types of cells play crucial roles in maintaining intestinal homeostasis. When microbial pathogens invade, intestinal epithelial cells (IECs) upregulate the expression of defensins immediately [27]. These defensins act like frontline warriors that directly combat invading pathogens. At the same time, alarm signals serve as signaling molecules, sending emergency alerts to nearby IECs and the immune system, triggering a more extensive defense response. For example, in the case of *Salmonella* infection, IECs can induce a substantial production of antimicrobial peptides (AMPs) and express pattern recognition receptors such as NLRP6, further enhancing intestinal defense capabilities [28]. It is noteworthy that ISCs also demonstrate remarkable adaptability in the face of infection. In certain situations, ISCs can change their fate determination and differentiate into more specialized defensive effector cells, such as enterocytes and Paneth cells. When *Salmonella* strikes, ISCs execute specific transcriptional programs to increase the number of enterocytes and Paneth cells, thus enhancing the effectiveness of infection resistance [28]. However, when faced with different pathogens, such as *Clostridioides difficile*, IECs may choose to self-sacrifice through apoptosis to prevent the spread of infection and protect the surrounding tissues [29]. In summary, different types of cells in the intestine collaboratively maintain the integrity of the intestinal epithelial barrier through complex interactions, thereby ensuring the homeostasis of the intestinal microecological system.

The unique cellular architecture of the intestine establishes a stable niche for commensal microorganisms. The major commensal bacterial communities in mammals consist of Gram-negative bacteria, such as those from the phylum Pseudomonadota and Bacteroidota, as well as Gram-positive bacteria from the phylum Bacillota [30]. Over millions of years of co-evolution, a mutualistic relationship has developed between the host and its commensal microbiota. Within the gut, commensal bacteria adapt to local niches to obtain nutrients and space while regulating the microbial community through resource competition, limiting invasive pathogens. [31]. In return, gut commensal bacteria provide a range of critical functions for the host, including promoting the integrity of the intestinal epithelial barrier and maintaining immune homeostasis [5]. However, when invasive pathogens (bacterium, virus, and helminth) enter the gut, this delicate balance may be disrupted, triggering host immune responses, which play a pivotal role in the development of gut health and disease.

## 3. Bacterial pathogen-induced immunity via intestinal epithelium

Intestinal homeostasis is maintained by a complex interplay among the epithelium, immune factors, and microbial flora, including bacteria, fungi, viruses, archaea, and protozoans [16]. During bacterial invasion, a cascade of signals is initiated that leads to the release of cytokines, chemokines, acute-phase proteins, and other immune effectors.

**Fig 1:**
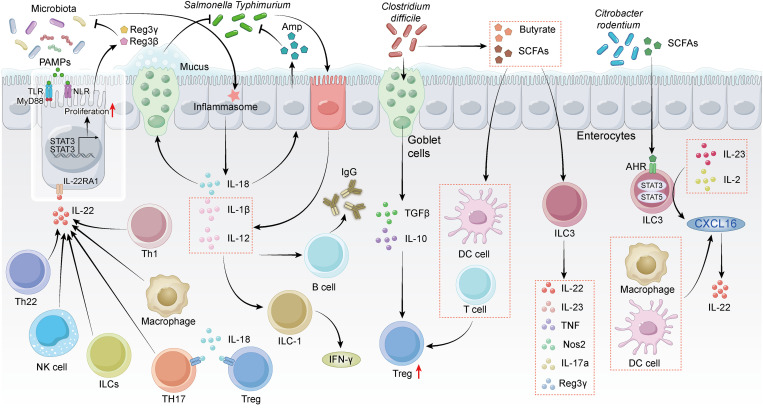
Immune responses to pathogens in intestinal epithelium. This diagram illustrates the immune responses initiated by pathogens in the intestinal epithelium, including interactions between microbiota, epithelial receptors (e.g., TLRs, NLRs), immune cells (e.g., ILCs, T cells, and macrophages), and cytokines (e.g., IL-22, IL-18, and IL-12). It highlights the coordination of innate and adaptive immunity in maintaining intestinal homeostasis and combating infections.

Cytokines from the IL-1 family, particularly IL-18, play a pivotal role in maintaining intestinal homeostasis during bacterial challenges (Fig 1). Released by intestinal epithelium, IL-18 relies on the activation of the inflammasome complex [32,33]. It is crucial for pathogen clearance, such as *Salmonella Typhimurium*, by inducing enterocytes to produce AMPs and goblet cells to secrete mucus [16]. Furthermore, IL-18 can influence CD4+ Th17 cells and Foxp3+ Tregs directly that express the IL-18R1 receptor, modulating their differentiation both in homeostasis and inflammatory states [34].

IL-22 plays an essential role in both innate and adaptive immune responses (Fig 1) [35]. It is secreted by T cells (Th1, Th17, Th22, CD8+ T cells, and γδ T cells), natural killer (NK) cells, and ILCs [36]. Induced by IL-6 and IL-23, Th1 and Th17 cells secrete IL-22, while TGF-β can inhibit its production [37–39]. Additionally, IL-12 and IL-17 can induce Th1 cells to produce IL-22 [36]. The accumulation of IL-22 activates the STAT3 signaling pathway, and then promoting the proliferation of IECs and stimulating the secretion of RegIIIβ and RegIIIγ [4]. *Salmonella* gastroenteritis infection induces the release of IL-1β and IL-12. Notably, IL-12 not only stimulates ILC1 to secrete IFN-γ but also promotes antibody production through a B-cell intrinsic IL-12/IFN-γ feed-forward loop. Meanwhile, IL-1β functions synergistically to enhance IFN-γ production by ILC1 [40–42].

While species from the *Clostridiales* family, such as *C. difficile*, invade IECs and induce goblet cells and CD103+ dendritic cells (DCs) to secrete TGF-β and IL-10, consequently promoting Treg proliferation. However, direct evidence demonstrating this mechanism’s contribution to immune evasion remains lacking [43–45]. Additionally, *Clostridium*-derived bioactive molecules, such as SCFAs and tryptophan, can activate DCs and T cells to induce Treg proliferation (Fig 1) [43]. However, in Rag1^−/−^ mice lacking T and B lymphocytes, ILC3s serve as the dominant immune responders during *Clostridium difficile* infection, showing upregulated expression of IL-22, IL-17a, and RegIIIγ. [41]. *Citrobacter rodentium* is a mouse-specific pathogen. In the initial stages of infection, ILC3s are activated through ligand-activated transcription factors, including aryl hydrocarbon receptor (AHR), RORγt, and Vitamin A and D receptors or bacteria-derived metabolites (SCFAs, Butyrate) [46–49]. The interaction among myeloid cells, such as CX3CR1+ DCs, TL1A+ macrophages, and ILC3s, mediates the secretion of the chemokine CXCL16, controlling the production of IL-22 and AMPs [50–52]. Mechanistically, STAT5a, STAT5b, and STAT3 signaling are initiated by IL-2 or IL-23, respectively [53]. Subsequently, IL-22 interacts with epithelial cells through the STAT3 signaling pathway to regulate immune responses, eliminate pathogens, and maintain intestinal homeostasis [4] (Fig 1).

## 4. Helminth-induced immunity via intestinal epithelium

Helminths can directly damage or activate IECs either through larval invasion or feeding [54,55]. Although the mechanisms by which the host sensing helminths are still not fully understood. The activation of type 2 immune responses is correlated with the release of alarmins, such as ATP, IL-25, IL-33, and thymic stromal lymphopoietin (TSLP), by epithelial cells [1,56].

**Fig 2:**
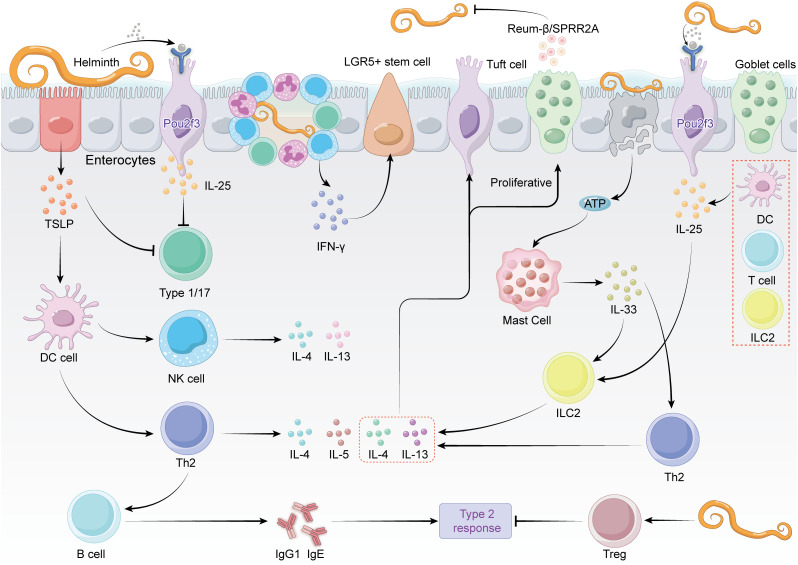
Immune responses to helminth in intestinal epithelium. This illustration depicts the immune response triggered by helminth infection in the intestinal epithelium. It highlights the role of tuft cells (producing IL-25), dendritic cells, and innate lymphoid cells (ILC2) in inducing Type 2 immunity. Key cytokines, including IL-4, IL-5, IL-13, and IL-33, drive the activation of Th2 cells, mast cells, and B-cells, leading to antibody production (IgG1/IgE) and helminth expulsion. Proliferative responses involving stem cells and communication with epithelial cells are also shown to maintain intestinal integrity.

Interleukin-33 (IL-33) plays an important role in regulating immune responses in epithelial cells (Fig 2). It helps maintain immune homeostasis and enhances defense against pathogens through promoting the release of Th2 cytokines, mediating tissue repair, and triggering inflammation [57]. IL-33 is produced by activated mast cells during *H. polygyrus* infection in response to ATP released from apoptotic epithelial cells [58]. In addition, IL-33 can induce ILC2 and Th2 cells to secret IL-13, which enhances the populations of tuft and goblet cells [59,60]. IL-33-deficient mice show defective type 2 responses and inefficient expulsion of *Nippostrongylus brasiliensis*, highlighting the importance of IL-33 in parasite clearance [61].

IL-25 (also known as IL-17E) is a unique cytokine within the IL-17 family, essential for inducing type 2 immune responses, maintaining tissue homeostasis, and stimulating injury repair (Fig 2) [62,63]. Produced by various immune cells such as T cells, DCs, and ILC2s, a significant source of IL-25 is tuft cells [59]. Tuft cells are crucial for recognizing helminth infections, and their absence in Pou2f3-deficient mice leads to reduced IL-25 production and defective type 2 responses [60]. During infection with *N. brasiliensis*, tuft cells significantly increase IL-25 production, recruiting CD4+ T cells and BATF-dependent ILC2s to the lamina propria [64]. The resulting production of IL-4 and IL-13 promotes the differentiation of tuft and goblet cells, facilitating mucus secretion and helminth expulsion [59,60].

TSLP was initially identified in mouse thymic stromal cells, and it plays an important role in the development and differentiation of lymphocytes (Fig 2) [65]. TSLP is produced by epithelial cells in response to helminth infections and binds to receptors on both intraepithelial immune cells and DCs. Upon activation by TSLP, DCs drive the polarization of Th2 responses, enhancing the expulsion of *T. muris* [1]. TSLP preferentially activates Th2 and Th9 cells, promoting the production of T2 cytokines while limiting pro-inflammatory cytokines, such as TNF-α, IL-1b, and IL-6, as well as Th1-polarizing cytokines (IL-12 and interferons). In addition, TSLP has been shown to suppress Th17-driven mucosal inflammation after bacterial colonization [66]. TSLP also stimulates NKT cells to produce IL-4 and IL-13, enhancing the type 2 immune response [67]. In the adaptive immune response, TSLP signaling directly influences naive T cells in the presence of T cell receptor stimulation, promoting the proliferation and differentiation of Th2 cells through the induction of IL-4 gene transcription [68–70]. Additionally, TSLP mediates the proliferation and differentiation of Tregs induced by DCs [71,72]. It also promotes B-cell lymphopoiesis by inducing the proliferation and differentiation of B-cell progenitors [73–75]. In the presence of TSLP, multilineage-committed CD34+ progenitor cells, pro B-cells, and pre B-cells differentiate and proliferate [76]. TSLP also induces the release of chemokines that attract T cells, including thymus and activation-regulated chemokine (TARC)/CCL17, DC-CK1/pulmonary and activation-regulated chemokine (PARC)/CCL18, macrophage-derived chemokine (MDC)/CCL22, and macrophage inflammatory protein (MIP3β)/CCL19.

## 5. Bacterial pathogen and commensal bacteria interactions

In the gut microbial ecosystem, commensal and pathogenic bacteria shape the host’s health-disease balance through complex ecological interactions involving nutrient competition, modulation of host immunity, and coexistence strategies [5]. Colonization resistance is a mechanism by which the gut microbiota prevents the colonization of pathogens through resource competition, niche occupation, and immune modulation [77]. Between the bacteria, colonization resistance can be influenced through producing inhibitory compounds or competing for resources. Additionally, commensal bacterial have indirect defensive mechanisms in the gut that restrict the invasion or expansion of pathogens. These include the microbiota-induced or microbiota-maintained mucus layer, oxygen levels, and both innate and adaptive immune responses [5].

### 5.1 Direct mechanisms

**Fig 3:**
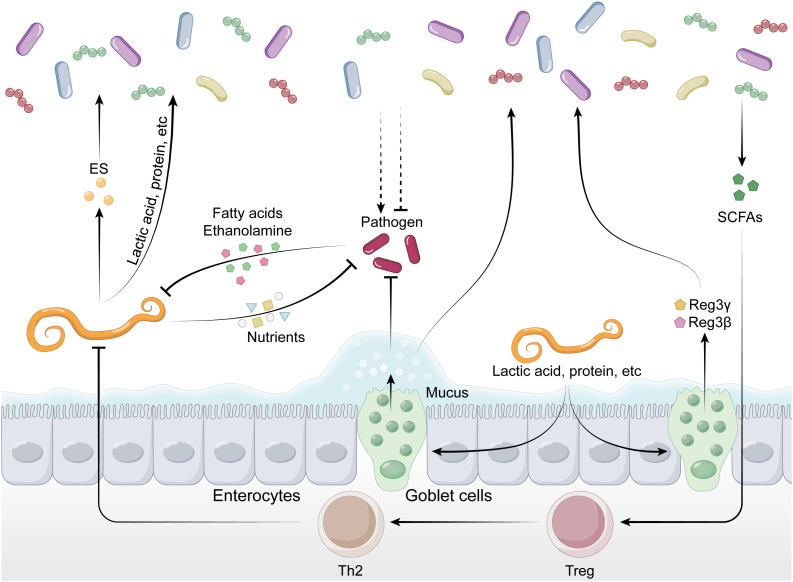
Competition and cooperation between bacterial pathogens and commensals. The illustration highlights the interplay between pathogens and commensals in the gut, involving nutrient competition, effector molecules, and metabolites. Commensals secrete bacteriocins, SCFAs, and metabolites to inhibit pathogens and strengthen the gut barrier. Pathogens, meanwhile, employ T3SS, T6SS, and induce reactive oxygen/nitrogen species (ROS/RNS) to invade host tissues. Additionally, the involvement of trace element chelation, antimicrobial peptides (AMPs), and the regulation of signaling molecules further contributes to the balance and resistance mechanisms of the microbial community.

The gut microbiota can restrict the expansion of pathogens by depleting the majority of available nutrients (Fig 3). For example, in germ-free (GF) mice or antibiotic-treated mice, the levels of sugars, amino acids, and other nutrients in the intestinal lumen increase, accompanied by a decline in colonization resistance [78–80]. Studies have shown that microbial consumption of dietary amino acids is a key factor in limiting the colonization of *S. Typhimurium*. Additionally, commensal bacteria mediate resistance to *S. Typhimurium* through the consumption of oxygen, iron, zinc, manganese, and anaerobic respiratory electron acceptors [81,82]. Beyond nutrient competition, certain active growth inhibition or infection mechanisms also promote colonization resistance. Bacteriocins are a heterogeneous class of peptides produced by bacteria, enhance colonization resistance by specifically inhibiting or killing targeted bacteria, typically those closely related to the producer, such as pathogenic Enterococci and other gram-positive bacteria [83–85]. In contrast, pheromones are small peptides secreted by Enterococci that specifically target pathogenic strains, such as virulent *Enterococcus faecalis*, to facilitate colonization resistance [86].

Gut bacteria employ contact-dependent growth inhibition system and secreted metabolites to compete and restrict pathogen colonization (Fig 3). A cell contact-dependent inhibition (CDI) system, termed CDI, was discovered in *E. coli* and later in other Pseudomonadota. CDI requires a specific receptor protein to recognize target cell and encode different toxic effector domains with various modes of inhibition. In addition, toxic effector genes are usually accompanied by a protective protein that neutralizes the toxin to protect the producing cell [87,88]. Another CDI system, the T6SS, can spear neighboring cells and inject toxic proteins without the need for a receptor. The system is widely present in Gram-negative bacteria, especially Pseudomonadota and Bacteroidota [88]. Beyond contact-dependent mechanisms, bacterial metabolites are also critical for colonization resistance. Secondary bile acids, generated through 7α/β-dehydroxylation by rare bacteria such as *Clostridium scindens*, inhibit Gram-positive bacteria like *C. difficile* and enhance resistance to infection [89–93]. Similarly, SCFAs, including acetate, propionate, and butyrate, can serve as an energy source for IECs and contribute to colonization resistance [94]. For example, SCFAs can suppress the growth of pathogenic *E. coli*, *C. rodentium*, and *C. difficile*, as well as limit the colonization of *S. Typhimurium* in the murine gut [95–98].

### 5.2 Indirect mechanisms

Besides directly inhibiting pathogen colonization, gut microbiota indirectly strengthen colonization resistance by preserving mucus layer, regulating oxygen levels, and modulating innate and adaptive immune responses.

The mucus layer forms a physical barrier that prevents pathogens from invading the underlying epithelium. For certain pathogens, such as *C. rodentium*, *Salmonella* species, and pathogenic *E. coli* strains, which rely on attachment to the epithelium to initiate infection and colonization, the mucus layer is particularly crucial in enhancing colonization resistance [5]. So far, studies have confirmed that a varied microbiota is important to promote mucus barrier function and colonization resistance in the presence of a disrupted mucus layer. For example, comparing the mucus layer between GF animals and conventionally raised mice reveals that GF animals have a thinner mucus layer, suggesting that microbial symbionts may enhance the mucosal barrier to limit pathogen colonization [99,100]. Similarly, mice lacking mucin 2 exhibit increased pathogen burden and greater disease severity following infection with *C. rodentium*, *S. Typhimurium*, and *Listeria monocytogenes* [101–103].

The gut microbiota also regulates oxygen levels within the intestine to inhibit pathogen expansion. For instance, members of the *Clostridia* class produce butyrate through β-oxidation, which enhances aerobic respiration in IECs, thereby depleting oxygen levels at the epithelial surface and restricting the growth of facultative anaerobes such as *S. Typhimurium* [104–107]. A depletion of *Clostridia* or a reduction in butyrate levels, often observed during inflammation and microbiota dysbiosis, elevates oxygen levels and facilitates pathogen proliferation. Additionally, commensal bacteria can compete for residual oxygen to limit pathogen colonization, such as by suppressing the expression of oxygen-dependent virulence genes in *Shigella flexneri* or restricting aerobic respiration in *C. rodentium* and *S. Typhimurium* [108–110]. Under anaerobic conditions, commensal bacteria such as *Mucispirillum schaedleri* further inhibit pathogen growth by competing for anaerobic respiratory substrates like nitrate, thereby limiting the expansion of pathogens that rely on nitrate respiration in the inflamed intestine [111–113]. This intricate regulation of oxygen levels and substrate competition underscores the pivotal role of gut microbiota in maintaining ecological balance and promoting colonization resistance against pathogens.

The gut microbiota inhibits pathogen colonization by stimulating the secretion of AMPs and proteins from host epithelial cells, exerting both antimicrobial effects and regulation of microbial balance. AMPs such as RegIIIβ and RegIIIγ, secreted by Paneth cells, maintain spatial segregation between commensals and IECs [114,115]. A reduction in these AMPs increases susceptibility to pathogen infection. For instance, microbial depletion by antibiotic administration reduces RegIIIγ expression, impairing the clearance of vancomycin-resistant *Enterococcus* [116]. Stimulation with Toll-like receptor 7 agonists or activation of the NOD1/2 signaling pathway can restore AMP production and limit pathogen colonization [117,118]. Lipocalin-2 (LCN2), another antimicrobial protein, inhibits pathogen growth by sequestering bacterial iron acquisition systems, and LCN2-deficient mice exhibit dysregulated gut microbiota composition [119–121]. Similarly, related proteins such as calprotectin demonstrate antimicrobial activity by chelating metal ions like iron, zinc, calcium, and manganese, although the relationship between calprotectin and the gut microbiota requires further investigation [122]. Collectively, these AMPs and proteins play critical roles in maintaining intestinal ecological balance and resisting pathogen colonization.

The gut microbiota also restricts pathogen colonization by inducing cytokines and adaptive immune responses. Microbiota-induced IL-22 enhances intestinal barrier function, alters microbiota composition, and promotes the growth of commensals like *Phascolarctobacterium*, which compete with pathogens, such as *C. rodentium* and *C. difficile* [123–125]. Additionally, IL-1β contributes to pathogen clearance, such as *S. Typhimurium* and *Pseudomonas aeruginosa*, by rapidly recruiting immune cells and neutrophils. In terms of adaptive immunity, certain commensals induce the production of polyreactive, low-affinity IgA antibodies, which cross-react with antigens expressed by gut pathogens and other bacterial species. Furthermore, IgA supports commensal expansion and maintains a favorable ecological niche, thereby limiting pathogen colonization [126–128]. Through these mechanisms, the gut microbiota collaborates with the immune system to provide resistance against intestinal pathogens and prevent their colonization.

### 5.3 Bacterial pathogen-driven counterattack

While being regulated by the gut microbiota, bacterial pathogens have evolved various strategies to evade colonization resistance (Fig 3). First, they employ virulence mechanisms to modify the intestinal environment for their own growth. For example, *S. Typhimurium* induces inflammation to produce reactive oxygen and nitrogen species, creating byproducts that can be utilized for respiration, while also exploiting the increased oxygen levels caused by inflammation to gain a growth advantage [3,129,130]. Additionally, *S. Typhimurium* uses high-affinity iron and zinc transport systems to outcompete other microbes for scarce metal resources. Second, pathogens directly antagonize the resident microbial community to establish ecological niches. The T6SS is a common competitive tool used by pathogens, which injects toxic effector molecules to kill neighboring bacteria [131]. For instance, *V. cholerae* leverages its T6SS to kill competing *E. coli*, enhancing its infectivity. Similarly, *S. Typhimurium* uses its T6SS to eliminate *Klebsiella oxytoca*, promoting its colonization. *Shigella sonnei* employs its T6SS to kill related species, such as *Shigella flexneri* and *E. coli*, thereby improving its ability to colonize. In addition, *C. rodentium* has been shown to utilize its T6SS during the early invasion stage to kill commensal *E. coli* or evade killing by other T6SS-bearing *E. coli*, enabling its successful colonization [132–134]. In addition, through the type III secretion system (T3SS), pathogens can inject effectors to alter the host’s physiological environment and promote their own growth. *C. rodentium* uses T3SS to adhere to IECs and inject effectors, inducing excessive epithelial proliferation, which releases oxygen for its respiration while metabolizing hydrogen peroxide (H_2_O_2_) to sustain growth [135]. *S. Typhimurium* employs T3SS effectors to trigger inflammatory responses, releasing reactive oxygen species and reactive nitrogen species that generate respiratory electron acceptors, such as tetrathionate (SO₄O_6_^2−^) and nitrate (NO_3_^−^), providing essential energy sources for its growth [5]. These mechanisms demonstrate that pathogens effectively overcome colonization resistance by altering the host environment and directly competing with the resident microbiota.

## 6. Helminth and commensal bacteria interactions

**Fig 4:**
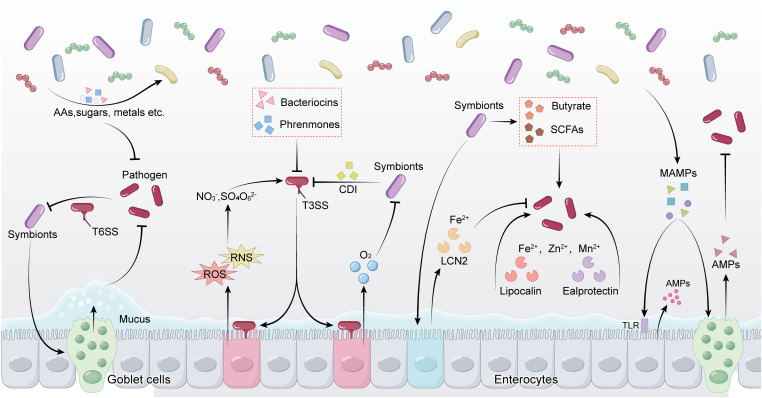
Competition and cooperation between helminths and commensals.

The influence of helminth infection on gut microbiota is intricate and multifaceted, occurring through diverse mechanisms (Fig 4). Helminth infections can affect the gene expression of intestinal bacteria, modulating their metabolic activities and survival capabilities [45]. While residing within the gut, helminths secrete various metabolic products that interact with gut bacteria, impacting bacterial growth and community structure [10]. Furthermore, there exists a competitive relationship between helminths and gut bacteria for nutritional resources, where the presence of helminths alters the nutrient environment within the gut, consequently influencing bacterial survival and reproduction [45]. In addition, helminth infection induces immune responses in the host, particularly Th2-type immune responses, which regulate the balance of the gut bacterial community. Helminth infection may also alter the physical and chemical environment of the gut, affecting microbial composition [56].

Helminths modulate gut microbiota by consuming nutrients and secreting excretory–secretory products (ES), while also stimulating goblet cells to produce mucus and antimicrobial peptides. They suppress pathogenic bacteria by competing for nutrients, whereas pathogens may inhibit helminth development by limiting fatty acids and ethanolamine. Meanwhile, commensal bacteria secrete short-chain fatty acids (SCFAs) to regulate Th2 immunity and restrict helminth infections. These interactions collectively shape and maintain gut microbial balance.So far, specific mechanisms of helminth and bacterial interactions are being elucidated. Continuous production and shedding of mucus play a significant role in blocking both helminth and bacterial infections. Beyond its defensive function, mucus also provides nutrition for certain commensal microorganisms [136]. The composition of mucus is regulated by glycosylation patterns, including the addition of sialic acid and sulfate residues [137]. Helminth infections induce Th2 immune responses that may regulate mucus modification through glycosylation and sulfation [138–140]. Additionally, the mucus-rich environment effectively prevents commensals, pathogens, and other large particulates from penetrating the intestinal epithelial monolayer into the mucosal and submucosal, while also serving as an anchor point for AMPs, including RegIIIγ and Relm-β to synergistically suppress pathogenic colonization. [1,141]. Numerous antimicrobial proteins (AMPs) have been identified, including a new one named small proline-rich protein 2A (SPRR2A), discovered during *H. polygyrus* infection in mice. SPRR2A is phylogenetically distinct from previously known AMPs [142]. Another intestinal bactericidal protein, resistin-like molecule β (RELMβ), primarily targets gram-negative bacteria and is dependent on type 2 cytokines [143]. Nutritional interactions are another significant aspect of helminth-bacterial dynamics. For instance, Venzon reported that *E. coli* mutants infected in gnotobiotic mice affected the proper development of *T. muris* parasites due to nutritional defects in fatty acid biosynthesis and ethanolamine utilization [144]. Helminth-induced intestinal epithelial damage caused by tissue-migrating larvae or adult worms can also modify bacterial communities by altering the intestinal environment [56]. In addition, the immune responses elicited by helminth infections can lead to various physiological alterations, influencing bacterial communities in the gut, including modifications in digesta transit time, which alters food digestion dynamics [145]. This altered digestion process is crucial for shaping both the composition and metabolic capabilities of the bacterial community.

The relationship between helminths and gut microbiota, shaped by evolution, is bidirectional. Recent studies have explored interactions between helminths and bacteria. In the case of *T. muris* infection in mice, diverse microbes are necessary for the optimal hatching of *T. muris* larvae from ingested eggs [8]. Conversely, structural changes in the cecal microbiome during infection can suppress subsequent parasite egg hatching, protecting the host from overcrowding [146]. Interestingly, many bacteria identified in animal studies modify the intestinal microbiota in both mice and pigs, utilizing chitin-based parasite egg shells as an energy source [147,148]. Additionally, Intestinal helminth larvae hatch in the soil and are known to carry bacteria into the host. Once inside, helminths may compete with intestinal bacteria for available nutrients (e.g., acetate and lactate) and/or secrete products (e.g., proteins and peptides) that alter bacterial growth [149,150]. Interestingly, it has been reported that protective bifidobacteria can also produce acetate, which in turn enhances epithelial cell-mediated intestinal defense, thereby protecting the host from fatal infections [6]. The presence or absence of the microbiota can also influence the colonization of helminths. For example, in animals treated with antibiotics or maintained in sterile environments, both larval and adult worms of *H. polygyrus* tend to reside closer to the duodenum, leading to reduced intestinal motility [151]. This significantly impedes the host’s critical role in the “weep and sweep” response against helminths [152]. Ultimately, both helminths and bacterial communities strive to establish long-term residence in the intestine without evoking inflammation [45].

## 7. Conclusions and perspectives

This article highlights the complex interactions among pathogenic bacteria, commensal microbiota, and helminths within the gut ecosystem. Commensal bacteria provide colonization resistance against pathogens through direct mechanisms, such as nutrient competition, production of inhibitory compounds, and secretion of metabolites like SCFAs and secondary bile acids. These metabolites not only inhibit pathogen growth but also support intestinal epithelial health. Indirectly, commensal bacteria regulate the gut environment by maintaining the mucus barrier, modulating oxygen levels, and inducing immune responses. However, pathogenic bacteria have evolved sophisticated strategies to counter colonization resistance, including the use of T6SS to kill commensal bacteria, inducing inflammation to alter the gut environment, and competing for resources such as iron and oxygen to establish ecological niches.

Meanwhile, helminths interact with commensal bacteria through both direct and indirect mechanisms, further complicating the gut ecosystem. Helminths secrete ES products that regulate bacterial gene expression, metabolic activity, and survival, while competing with bacteria for nutritional resources, thereby altering the composition and functionality of the microbiota. Indirectly, helminth infections elicit Th2 immune responses that regulate microbial balance through cytokines (e.g., IL-18 and IL-22) and antimicrobial proteins (e.g., SPRR2A and RELMβ). These immune responses reshape the gut environment, promoting the expansion of anti-inflammatory bacterial populations and enhancing the mucus barrier to support the growth of beneficial bacteria. Interestingly, helminths and pathogenic bacteria may exhibit either synergistic or competitive relationships. For example, helminths may provide a survival niche for pathogenic bacteria, as observed in the colonization of *V. cholerae* within the intestines of *Ascaris lumbricoides*. Conversely, helminths may indirectly inhibit pathogenic bacteria through immune modulation.

Currently, studies on the mechanisms underlying interactions among commensal bacteria, pathogens, and helminths remain limited. This raises several important questions worth exploring. For instance, does the disruption of intestinal epithelial tight junctions by pathogenic bacteria via the T3SS facilitate helminth infection? If so, what are the specific mechanisms? Furthermore, how do pathogenic bacteria sense changes in the gut microbiota or metabolite composition through two-component systems? How does this sensing regulate gene expression to promote infection and colonization? During helminth infections, do changes in gut commensal bacterial metabolites influence the virulence of pathogens? If so, through which metabolic or signaling pathways? In addition, bacterial T6SS can kill competing bacteria within the same niche by secreting toxins. Could helminths exploit certain gut bacteria to utilize T6SS, eliminating competitors and obtaining resources to establish ecological dominance?

Helminth infections also impact the gut environment, but whether these effects are beneficial or detrimental remains unclear. What mechanisms mediate these outcomes? For example, helminth infection induces a Th2-skewed immune response, including cytokines such as IL-4 and IL-10. This suppresses Th1 responses, including IFN-γ, thereby weakening host cellular immunity and reducing resistance to intracellular pathogens like *Salmonella* and *Mycobacterium*. On the other hand, helminths may regulate gut microbiota by increasing beneficial bacterial populations or reducing the colonization of pathogens such as *E. coli*. Additionally, helminths secrete anti-inflammatory molecules, which alleviate inflammation and restore gut homeostasis. These mechanisms may interact with one another, further complicating the dynamic relationships within this system.

**Fig 5:**
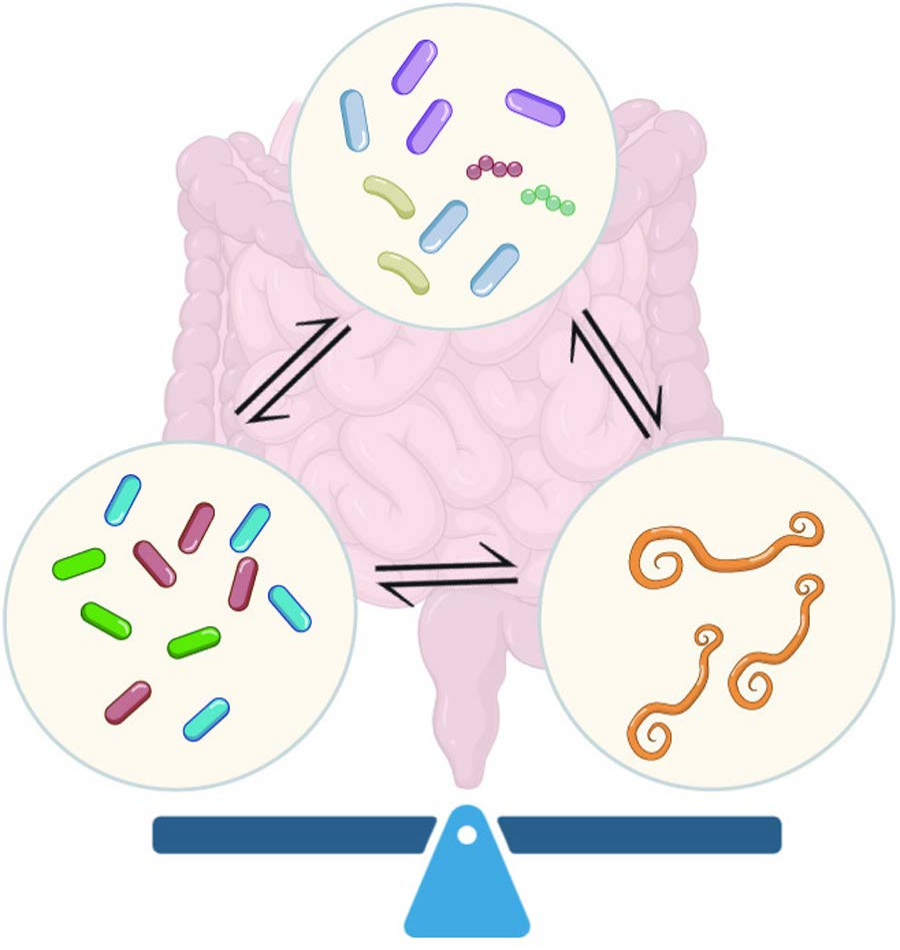
The balance among bacterial pathogens, helminths, and commensal bacteria.

Understanding how commensal bacteria, pathogens, and helminths interact to orchestrate intestinal homeostasis is crucial (Fig 5). Such insights could deepen our understanding of microecological regulation within the host and provide a theoretical foundation for developing effective strategies to prevent and manage intestinal diseases.

## References

[1] Coakley G, Harris NL. The intestinal epithelium at the forefront of host–helminth interactions. Trends Parasitol. 2020;36(9):761–72. doi: 10.1016/j.pt.2020.07.002 32713764

[2] Rooks MG, Garrett WS. Gut microbiota, metabolites and host immunity. Nat Rev Immunol. 2016;16(6):341–52. doi: 10.1038/nri.2016.42 27231050 PMC5541232

[3] Winter SE, Thiennimitr P, Winter MG, Butler BP, Huseby DL, Crawford RW, et al. Gut inflammation provides a respiratory electron acceptor for *Salmonella*. Nature. 2010;467(7314):426–9. doi: 10.1038/nature09415 20864996 PMC2946174

[4] Zheng Y, Valdez PA, Danilenko DM, Hu Y, Sa SM, Gong Q, et al. Interleukin-22 mediates early host defense against attaching and effacing bacterial pathogens. Nat Med. 2008;14(3):282–9. doi: 10.1038/nm1720 18264109

[5] Caballero-Flores G, Pickard JM, Núñez G. Microbiota-mediated colonization resistance: mechanisms and regulation. Nat Rev Microbiol. 2023;21(6):347–60. doi: 10.1038/s41579-022-00833-7 36539611 PMC10249723

[6] Fukuda S, Toh H, Hase K, Oshima K, Nakanishi Y, Yoshimura K, et al. Bifidobacteria can protect from enteropathogenic infection through production of acetate. Nature. 2011;469(7331):543–7. doi: 10.1038/nature09646 21270894

[7] Salgame P, Yap GS, Gause WC. Effect of helminth-induced immunity on infections with microbial pathogens. Nat Immunol. 2013;14(11):1118–26. doi: 10.1038/ni.2736 24145791 PMC4955540

[8] Hayes KS, Bancroft AJ, Goldrick M, Portsmouth C, Roberts IS, Grencis RK. Exploitation of the intestinal microflora by the parasitic nematode *Trichuris muris*. Science. 2010;328(5984):1391–4. doi: 10.1126/science.1187703 20538949 PMC3428897

[9] Reynolds LA, Smith KA, Filbey KJ, Harcus Y, Hewitson JP, Redpath SA, et al. Commensal-pathogen interactions in the intestinal tract: lactobacilli promote infection with, and are promoted by, helminth parasites. Gut Microbes. 2014;5(4):522–32. doi: 10.4161/gmic.32155 25144609 PMC4822684

[10] Midha A, Janek K, Niewienda A, Henklein P, Guenther S, Serra DO, et al. Corrigendum: the intestinal roundworm *Ascaris suum* releases antimicrobial factors which interfere with bacterial growth and biofilm formation. Front Cell Infect Microbiol. 2018;8:367. doi: 10.3389/fcimb.2018.00367 30364807 PMC6198275

[11] Rausch S, Midha A, Kuhring M, Affinass N, Radonic A, Kühl AA, et al. Parasitic nematodes exert antimicrobial activity and benefit from microbiota-driven support for host immune regulation. Front Immunol. 2018;9:2282. doi: 10.3389/fimmu.2018.02282 30349532 PMC6186814

[12] Midha A, Ebner F, Schlosser-Brandenburg J, Rausch S, Hartmann S. Trilateral relationship: ascaris, microbiota, and host cells. Trends Parasitol. 2021;37(3):251–62. doi: 10.1016/j.pt.2020.09.002 33008723

[13] Midha A, Schlosser J, Hartmann S. Reciprocal interactions between nematodes and their microbial environments. Front Cell Infect Microbiol. 2017;7:144. doi: 10.3389/fcimb.2017.00144 28497029 PMC5406411

[14] Nalin DR, McLaughlin J. Colonization of *Ascaris lumbricoides* by *V. cholerae*. J Parasitol. 1976;62(5):839–41. doi: 10.2307/3278979 978375

[15] Peterson LW, Artis D. Intestinal epithelial cells: regulators of barrier function and immune homeostasis. Nat Rev Immunol. 2014;14(3):141–53. doi: 10.1038/nri3608 24566914

[16] Mahapatro M, Erkert L, Becker C. Cytokine-mediated crosstalk between immune cells and epithelial cells in the gut. Cells. 2021;10(1):111. doi: 10.3390/cells10010111 33435303 PMC7827439

[17] Owens BMJ, Simmons A. Intestinal stromal cells in mucosal immunity and homeostasis. Mucosal Immunol. 2013;6(2):224–34. doi: 10.1038/mi.2012.125 23235744

[18] Palm NW, Rosenstein RK, Medzhitov R. Allergic host defences. Nature. 2012;484(7395):465–72. doi: 10.1038/nature11047 22538607 PMC3596087

[19] Loke P, Cadwell K. Getting a taste for parasites in the gut. Immunity. 2018;49(1):16–8. doi: 10.1016/j.immuni.2018.07.002 30021142

[20] Hoerauf A, Satoguina J, Saeftel M, Specht S. Immunomodulation by filarial nematodes. Parasite Immunol. 2005;27(10–11):417–29. doi: 10.1111/j.1365-3024.2005.00792.x 16179035

[21] Anthony RM, Rutitzky LI, Urban JF Jr, Stadecker MJ, Gause WC. Protective immune mechanisms in helminth infection. Nat Rev Immunol. 2007;7(12):975–87. doi: 10.1038/nri2199 18007680 PMC2258092

[22] Allen JE, Maizels RM. Diversity and dialogue in immunity to helminths. Nat Rev Immunol. 2011;11(6):375–88. doi: 10.1038/nri2992 21610741

[23] Samuel S, Walsh R, Webb J, Robins A, Potten C, Mahida YR. Characterization of putative stem cells in isolated human colonic crypt epithelial cells and their interactions with myofibroblasts. Am J Physiol Cell Physiol. 2009;296(2):C296–305. doi: 10.1152/ajpcell.00383.2008 19073897 PMC2643851

[24] Farin HF, Van Es JH, Clevers H. Redundant sources of Wnt regulate intestinal stem cells and promote formation of Paneth cells. Gastroenterol. 2012;143(6):1518-1529.e7. doi: 10.1053/j.gastro.2012.08.031 22922422

[25] Brown SL, Riehl TE, Walker MR, Geske MJ, Doherty JM, Stenson WF, et al. Myd88-dependent positioning of Ptgs2-expressing stromal cells maintains colonic epithelial proliferation during injury. J Clin Invest. 2007;117(1):258–69. doi: 10.1172/JCI29159 17200722 PMC1716207

[26] Groschwitz KR, Hogan SP. Intestinal barrier function: molecular regulation and disease pathogenesis. J Allergy Clin Immunol. 2009;124(1):3–20; quiz 21–2. doi: 10.1016/j.jaci.2009.05.038 19560575 PMC4266989

[27] Larsen SB, Cowley CJ, Fuchs E. Epithelial cells: liaisons of immunity. Curr Opin Immunol. 2020;62:45–53. doi: 10.1016/j.coi.2019.11.004 31874430 PMC7067656

[28] Haber AL, Biton M, Rogel N, Herbst RH, Shekhar K, Smillie C, et al. A single-cell survey of the small intestinal epithelium. Nature. 2017;551(7680):333–9. doi: 10.1038/nature24489 29144463 PMC6022292

[29] Saavedra PHV, Huang L, Ghazavi F, Kourula S, Vanden Berghe T, Takahashi N, et al. Apoptosis of intestinal epithelial cells restricts *Clostridium difficile* infection in a model of pseudomembranous colitis. Nat Commun. 2018;9(1):4846. doi: 10.1038/s41467-018-07386-5 30451870 PMC6242954

[30] NIH HMP Working Group, Peterson J, Garges S, Giovanni M, McInnes P, Wang L, et al. The NIH human microbiome project. Genome Res. 2009;19(12):2317–23. doi: 10.1101/gr.096651.109 19819907 PMC2792171

[31] Pickard JM, Zeng MY, Caruso R, Núñez G. Gut microbiota: role in pathogen colonization, immune responses, and inflammatory disease. Immunol Rev. 2017;279(1):70–89. doi: 10.1111/imr.12567 28856738 PMC5657496

[32] Nowarski R, Jackson R, Gagliani N, de Zoete MR, Palm NW, Bailis W, et al. Epithelial IL-18 equilibrium controls barrier function in colitis. Cell. 2015;163(6):1444–56. doi: 10.1016/j.cell.2015.10.072 26638073 PMC4943028

[33] Palomo J, Dietrich D, Martin P, Palmer G, Gabay C. The interleukin (IL)-1 cytokine family—balance between agonists and antagonists in inflammatory diseases. Cytokine. 2015;76(1):25–37. doi: 10.1016/j.cyto.2015.06.017 26185894

[34] Harrison OJ, Srinivasan N, Pott J, Schiering C, Krausgruber T, Ilott NE, et al. Epithelial-derived IL-18 regulates Th17 cell differentiation and Foxp3⁺ Treg cell function in the intestine. Mucosal Immunol. 2015;8(6):1226–36. doi: 10.1038/mi.2015.13 25736457 PMC4368110

[35] Shohan M, Dehghani R, Khodadadi A, Dehnavi S, Ahmadi R, Joudaki N, et al. Interleukin-22 and intestinal homeostasis: protective or destructive? IUBMB Life. 2020;72(8):1585–602. doi: 10.1002/iub.2295 32365282

[36] Satoh-Takayama N, Vosshenrich CAJ, Lesjean-Pottier S, Sawa S, Lochner M, Rattis F, et al. Microbial flora drives interleukin 22 production in intestinal NKp46+ cells that provide innate mucosal immune defense. Immunity. 2008;29(6):958–70. doi: 10.1016/j.immuni.2008.11.001 19084435

[37] Zheng Y, Danilenko DM, Valdez P, Kasman I, Eastham-Anderson J, Wu J, et al. Interleukin-22, a T(H)17 cytokine, mediates IL-23-induced dermal inflammation and acanthosis. Nature. 2007;445(7128):648–51. doi: 10.1038/nature05505 17187052

[38] Liang SC, Tan X-Y, Luxenberg DP, Karim R, Dunussi-Joannopoulos K, Collins M, et al. Interleukin (IL)-22 and IL-17 are coexpressed by Th17 cells and cooperatively enhance expression of antimicrobial peptides. J Exp Med. 2006;203(10):2271–9. doi: 10.1084/jem.20061308 16982811 PMC2118116

[39] Rutz S, Noubade R, Eidenschenk C, Ota N, Zeng W, Zheng Y, et al. Transcription factor c-Maf mediates the TGF-β-dependent suppression of IL-22 production in T(H)17 cells. Nat Immunol. 2011;12(12):1238–45. doi: 10.1038/ni.2134 22001828

[40] Klose CSN, Kiss EA, Schwierzeck V, Ebert K, Hoyler T, d’Hargues Y, et al. A T-bet gradient controls the fate and function of CCR6-RORγt^+^ innate lymphoid cells. Nature. 2013;494(7436):261–5. doi: 10.1038/nature11813 23334414

[41] Beck K, Ohno H, Satoh-Takayama N. Innate lymphoid cells: important regulators of host–bacteria interaction for border defense. Microorganisms. 2020;8(9):1342. doi: 10.3390/microorganisms8091342 32887435 PMC7563982

[42] Elsner RA, Smita S, Shlomchik MJ. IL-12 induces a B cell-intrinsic IL-12/IFNγ feed-forward loop promoting extrafollicular B cell responses. Nat Immunol. 2024;25(7):1283–95. doi: 10.1038/s41590-024-01858-1 38862796 PMC11992614

[43] Brown EM, Kenny DJ, Xavier RJ. Gut microbiota regulation of T cells during inflammation and autoimmunity. Annu Rev Immunol. 2019;37:599–624. doi: 10.1146/annurev-immunol-042718-041841 31026411

[44] Wang L, Villafuerte Gálvez JA, Lee C, Wu S, Kelly CP, Chen X, et al. Understanding host immune responses in *Clostridioides difficile* infection: implications for pathogenesis and immunotherapy. Imeta. 2024;3(3):e200. doi: 10.1002/imt2.200 38898983 PMC11183162

[45] Loke P, Harris NL. Networking between helminths, microbes, and mammals. Cell Host Microbe. 2023;31(4):464–71. doi: 10.1016/j.chom.2023.02.008 37054669 PMC10273824

[46] Kiss EA, Vonarbourg C, Kopfmann S, Hobeika E, Finke D, Esser C, et al. Natural aryl hydrocarbon receptor ligands control organogenesis of intestinal lymphoid follicles. Science. 2011;334(6062):1561–5. doi: 10.1126/science.1214914 22033518

[47] Chen J, Waddell A, Lin Y-D, Cantorna MT. Dysbiosis caused by vitamin D receptor deficiency confers colonization resistance to *Citrobacter rodentium* through modulation of innate lymphoid cells. Mucosal Immunol. 2015;8(3):618–26. doi: 10.1038/mi.2014.94 25315967 PMC4398576

[48] Yang W, Yu T, Huang X, Bilotta AJ, Xu L, Lu Y, et al. Intestinal microbiota-derived short-chain fatty acids regulation of immune cell IL-22 production and gut immunity. Nat Commun. 2020;11(1):4457. doi: 10.1038/s41467-020-18262-6 32901017 PMC7478978

[49] Hanlon N, Gillan N, Neil J, Seidler K. The role of the aryl hydrocarbon receptor (AhR) in modulating intestinal ILC3s to optimise gut pathogen resistance in lupus and benefits of nutritional AhR ligands. Clin Nutr. 2024;43(6):1199–215. doi: 10.1016/j.clnu.2024.04.008 38631087

[50] Manta C, Heupel E, Radulovic K, Rossini V, Garbi N, Riedel CU, et al. CX(3)CR1(+) macrophages support IL-22 production by innate lymphoid cells during infection with *Citrobacter rodentium*. Mucosal Immunol. 2013;6(1):177–88. doi: 10.1038/mi.2012.61 22854708 PMC3534171

[51] Longman RS, Diehl GE, Victorio DA, Huh JR, Galan C, Miraldi ER, et al. CX₃CR1⁺ mononuclear phagocytes support colitis-associated innate lymphoid cell production of IL-22. J Exp Med. 2014;211(8):1571–83. doi: 10.1084/jem.20140678 25024136 PMC4113938

[52] Satoh-Takayama N, Serafini N, Verrier T, Rekiki A, Renauld J-C, Frankel G, et al. The chemokine receptor CXCR6 controls the functional topography of interleukin-22 producing intestinal innate lymphoid cells. Immunity. 2014;41(5):776–88. doi: 10.1016/j.immuni.2014.10.007 25456160

[53] Bauché D, Joyce-Shaikh B, Fong J, Villarino AV, Ku KS, Jain R, et al. IL-23 and IL-2 activation of STAT5 is required for optimal IL-22 production in ILC3s during colitis. Sci Immunol. 2020;5(46):eaav1080. doi: 10.1126/sciimmunol.aav1080 32332067

[54] Madden KB, Yeung KA, Zhao A, Gause WC, Finkelman FD, Katona IM, et al. Enteric nematodes induce stereotypic STAT6-dependent alterations in intestinal epithelial cell function. J Immunol. 2004;172(9):5616–21. doi: 10.4049/jimmunol.172.9.5616 15100305

[55] Su C, Cao Y, Kaplan J, Zhang M, Li W, Conroy M, et al. Duodenal helminth infection alters barrier function of the colonic epithelium via adaptive immune activation. Infect Immun. 2011;79(6):2285–94. doi: 10.1128/IAI.01123-10 21444669 PMC3125859

[56] Rapin A, Harris NL. Helminth-bacterial interactions: cause and consequence. Trends Immunol. 2018;39(9):724–33. doi: 10.1016/j.it.2018.06.002 29941203

[57] Zhou Z, Yan F, Liu O. Interleukin (IL)-33: an orchestrator of immunity from host defence to tissue homeostasis. Clin Transl Immunol. 2020;9(6):e1146. doi: 10.1002/cti2.1146 32566227 PMC7299676

[58] Shimokawa C, Kanaya T, Hachisuka M, Ishiwata K, Hisaeda H, Kurashima Y, et al. Mast cells are crucial for induction of group 2 innate lymphoid cells and clearance of helminth infections. Immunity. 2017;46(5):863-874.e4. doi: 10.1016/j.immuni.2017.04.017 28514691

[59] Gerbe F, Sidot E, Smyth DJ, Ohmoto M, Matsumoto I, Dardalhon V, et al. Intestinal epithelial tuft cells initiate type 2 mucosal immunity to helminth parasites. Nature. 2016;529(7585):226–30. doi: 10.1038/nature16527 26762460 PMC7614903

[60] von Moltke J, Ji M, Liang H-E, Locksley RM. Tuft-cell-derived IL-25 regulates an intestinal ILC2-epithelial response circuit. Nature. 2016;529(7585):221–5. doi: 10.1038/nature16161 26675736 PMC4830391

[61] Hung L-Y, Lewkowich IP, Dawson LA, Downey J, Yang Y, Smith DE, et al. IL-33 drives biphasic IL-13 production for noncanonical Type 2 immunity against hookworms. Proc Natl Acad Sci U S A. 2013;110(1):282–7. doi: 10.1073/pnas.1206587110 23248269 PMC3538196

[62] Owyang AM, Zaph C, Wilson EH, Guild KJ, McClanahan T, Miller HRP, et al. Interleukin 25 regulates type 2 cytokine-dependent immunity and limits chronic inflammation in the gastrointestinal tract. J Exp Med. 2006;203(4):843–9. doi: 10.1084/jem.20051496 16606667 PMC1800834

[63] Borowczyk J, Shutova M, Brembilla NC, Boehncke W-H. IL-25 (IL-17E) in epithelial immunology and pathophysiology. J Allergy Clin Immunol. 2021;148(1):40–52. doi: 10.1016/j.jaci.2020.12.628 33485651

[64] Miller MM, Patel PS, Bao K, Danhorn T, O’Connor BP, Reinhardt RL. BATF acts as an essential regulator of IL-25-responsive migratory ILC2 cell fate and function. Sci Immunol. 2020;5(43):eaay3994. doi: 10.1126/sciimmunol.aay3994 31924686 PMC7112987

[65] Sims JE, Williams DE, Morrissey PJ, Garka K, Foxworthe D, Price V, et al. Molecular cloning and biological characterization of a novel murine lymphoid growth factor. J Exp Med. 2000;192(5):671–80. doi: 10.1084/jem.192.5.671 10974033 PMC2193273

[66] Grencis RK, Worthington JJ. Tuft cells: a new flavor in innate epithelial immunity. Trends Parasitol. 2016;32(8):583–5. doi: 10.1016/j.pt.2016.04.016 27161767

[67] Wu WH, Park CO, Oh SH, Kim HJ, Kwon YS, Bae BG, et al. Thymic stromal lymphopoietin-activated invariant natural killer T cells trigger an innate allergic immune response in atopic dermatitis. J Allergy Clin Immunol. 2010;126(2):290–9, 299.e1-4. doi: 10.1016/j.jaci.2010.05.024 20624642

[68] Rochman Y, Dienger-Stambaugh K, Richgels PK, Lewkowich IP, Kartashov AV, Barski A, et al. TSLP signaling in CD4^+^ T cells programs a pathogenic T helper 2 cell state. Sci Signal. 2018;11(521):eaam8858. doi: 10.1126/scisignal.aam8858 29535264 PMC6039124

[69] Omori M, Ziegler S. Induction of IL-4 expression in CD4(+) T cells by thymic stromal lymphopoietin. J Immunol. 2007;178(3):1396–404. doi: 10.4049/jimmunol.178.3.1396 17237387

[70] Kitajima M, Lee H-C, Nakayama T, Ziegler SF. TSLP enhances the function of helper type 2 cells. Eur J Immunol. 2011;41(7):1862–71. doi: 10.1002/eji.201041195 21484783 PMC3124605

[71] Li H, Zhao H, Yu J, Su Y, Cao S, An X, et al. Increased prevalence of regulatory T cells in the lung cancer microenvironment: a role of thymic stromal lymphopoietin. Cancer Immunol Immunother. 2011;60(11):1587–96. doi: 10.1007/s00262-011-1059-6 21681373 PMC11028680

[72] Watanabe N, Hanabuchi S, Soumelis V, Yuan W, Ho S, de Waal Malefyt R, et al. Human thymic stromal lymphopoietin promotes dendritic cell-mediated CD4+ T cell homeostatic expansion. Nat Immunol. 2004;5(4):426–34. doi: 10.1038/ni1048 14991051

[73] Levin SD, Koelling RM, Friend SL, Isaksen DE, Ziegler SF, Perlmutter RM, et al. Thymic stromal lymphopoietin: a cytokine that promotes the development of IgM+ B cells in vitro and signals via a novel mechanism. J Immunol. 1999;162(2):677–83. doi: 10.4049/jimmunol.162.2.677 9916685

[74] Friend SL, Hosier S, Nelson A, Foxworthe D, Williams DE, Farr A. A thymic stromal cell line supports in vitro development of surface IgM+ B cells and produces a novel growth factor affecting B and T lineage cells. Exp Hematol. 1994;22(3):321–8. 8112430

[75] Milford T-AM, Su RJ, Francis OL, Baez I, Martinez SR, Coats JS, et al. TSLP or IL-7 provide an IL-7Rα signal that is critical for human B lymphopoiesis. Eur J Immunol. 2016;46(9):2155–61. doi: 10.1002/eji.201646307 27325567 PMC5056642

[76] Scheeren FA, van Lent AU, Nagasawa M, Weijer K, Spits H, Legrand N, et al. Thymic stromal lymphopoietin induces early human B-cell proliferation and differentiation. Eur J Immunol. 2010;40(4):955–65. doi: 10.1002/eji.200939419 20127673

[77] van der Waaij D, Berghuis-de Vries JM, Lekkerkerk Lekkerkerk-v. Colonization resistance of the digestive tract in conventional and antibiotic-treated mice. J Hyg (Lond). 1971;69(3):405–11. doi: 10.1017/s0022172400021653 4999450 PMC2130899

[78] BOHNHOFF M, DRAKE BL, MILLER CP. Effect of streptomycin on susceptibility of intestinal tract to experimental *Salmonella* infection. Proc Soc Exp Biol Med. 1954;86(1):132–7. doi: 10.3181/00379727-86-21030 13177610

[79] Caballero-Flores G, Pickard JM, Fukuda S, Inohara N, Núñez G. An enteric pathogen subverts colonization resistance by evading competition for amino acids in the gut. Cell Host Microbe. 2020;28(4):526-533.e5. doi: 10.1016/j.chom.2020.06.018 32726577 PMC7554178

[80] Theriot CM, Koenigsknecht MJ, Carlson PE Jr, Hatton GE, Nelson AM, Li B, et al. Antibiotic-induced shifts in the mouse gut microbiome and metabolome increase susceptibility to *Clostridium difficile* infection. Nat Commun. 2014;5:3114. doi: 10.1038/ncomms4114 24445449 PMC3950275

[81] Deriu E, Liu JZ, Pezeshki M, Edwards RA, Ochoa RJ, Contreras H, et al. Probiotic bacteria reduce *Salmonella Typhimurium* intestinal colonization by competing for iron. Cell Host Microbe. 2013;14(1):26–37. doi: 10.1016/j.chom.2013.06.007 23870311 PMC3752295

[82] Behnsen J, Zhi H, Aron AT, Subramanian V, Santus W, Lee MH, et al. Siderophore-mediated zinc acquisition enhances enterobacterial colonization of the inflamed gut. Nat Commun. 2021;12(1):7016. doi: 10.1038/s41467-021-27297-2 34853318 PMC8636617

[83] Heilbronner S, Krismer B, Brötz-Oesterhelt H, Peschel A. The microbiome-shaping roles of bacteriocins. Nat Rev Microbiol. 2021;19(11):726–39. doi: 10.1038/s41579-021-00569-w 34075213

[84] Gillor O, Giladi I, Riley MA. Persistence of colicinogenic *Escherichia coli* in the mouse gastrointestinal tract. BMC Microbiol. 2009;9:165. doi: 10.1186/1471-2180-9-165 19674447 PMC2741469

[85] Caballero S, Kim S, Carter RA, Leiner IM, Sušac B, Miller L, et al. Cooperating commensals restore colonization resistance to vancomycin-resistant *Enterococcus faecium*. Cell Host Microbe. 2017;21(5):592-602.e4. doi: 10.1016/j.chom.2017.04.002 28494240 PMC5494988

[86] Kim SG, Becattini S, Moody TU, Shliaha PV, Littmann ER, Seok R, et al. Microbiota-derived lantibiotic restores resistance against vancomycin-resistant *Enterococcus*. Nature. 2019;572(7771):665–9. doi: 10.1038/s41586-019-1501-z 31435014 PMC6717508

[87] Hayes CS, Koskiniemi S, Ruhe ZC, Poole SJ, Low DA. Mechanisms and biological roles of contact-dependent growth inhibition systems. Cold Spring Harb Perspect Med. 2014;4(2):a010025. doi: 10.1101/cshperspect.a010025 24492845 PMC3904093

[88] Coyne MJ, Roelofs KG, Comstock LE. Type VI secretion systems of human gut Bacteroidales segregate into three genetic architectures, two of which are contained on mobile genetic elements. BMC Genomics. 2016;17:58. doi: 10.1186/s12864-016-2377-z 26768901 PMC4714493

[89] Guzior DV, Quinn RA. Review: microbial transformations of human bile acids. Microbiome. 2021;9(1):140. doi: 10.1186/s40168-021-01101-1 34127070 PMC8204491

[90] Sannasiddappa TH, Lund PA, Clarke SR. *In vitro* antibacterial activity of unconjugated and conjugated bile salts on *Staphylococcus aureus*. Front Microbiol. 2017;8:1581. doi: 10.3389/fmicb.2017.01581 28878747 PMC5572772

[91] Watanabe M, Fukiya S, Yokota A. Comprehensive evaluation of the bactericidal activities of free bile acids in the large intestine of humans and rodents. J Lipid Res. 2017;58(6):1143–52. doi: 10.1194/jlr.M075143 28404640 PMC5454512

[92] Sun X, Winglee K, Gharaibeh RZ, Gauthier J, He Z, Tripathi P, et al. Microbiota-derived metabolic factors reduce campylobacteriosis in mice. Gastroenterology. 2018;154(6):1751-1763.e2. doi: 10.1053/j.gastro.2018.01.042 29408609 PMC5927838

[93] Buffie CG, Bucci V, Stein RR, McKenney PT, Ling L, Gobourne A, et al. Precision microbiome reconstitution restores bile acid mediated resistance to *Clostridium difficile*. Nature. 2015;517(7533):205–8. doi: 10.1038/nature13828 25337874 PMC4354891

[94] Sorbara MT, Dubin K, Littmann ER, Moody TU, Fontana E, Seok R, et al. Inhibiting antibiotic-resistant Enterobacteriaceae by microbiota-mediated intracellular acidification. J Exp Med. 2019;216(1):84–98. doi: 10.1084/jem.20181639 30563917 PMC6314524

[95] Bohnhoff M, Miller CP, Martin WR. Resistance of the mouse’s intestinal tract to experimental *Salmonella infection*: II. Factors responsibie for its loss following streptomycin treatment. J Exp Med. 1964;120(5):817–28. doi: 10.1084/jem.120.5.817 14247722 PMC2137859

[96] Shin R, Suzuki M, Morishita Y. Influence of intestinal anaerobes and organic acids on the growth of enterohaemorrhagic *Escherichia coli* O157:H7. J Med Microbiol. 2002;51(3):201–6. doi: 10.1099/0022-1317-51-3-201 11871614

[97] Su WJ, Waechter MJ, Bourlioux P, Dolegeal M, Fourniat J, Mahuzier G. Role of volatile fatty acids in colonization resistance to *Clostridium difficile* in gnotobiotic mice. Infect Immun. 1987;55(7):1686–91. doi: 10.1128/iai.55.7.1686-1691.1987 3596806 PMC260579

[98] Osbelt L, Thiemann S, Smit N, Lesker TR, Schröter M, Gálvez EJC, et al. Variations in microbiota composition of laboratory mice influence *Citrobacter rodentium* infection via variable short-chain fatty acid production. PLoS Pathog. 2020;16(3):e1008448. doi: 10.1371/journal.ppat.1008448 32208465 PMC7141690

[99] Petersson J, Schreiber O, Hansson GC, Gendler SJ, Velcich A, Lundberg JO, et al. Importance and regulation of the colonic mucus barrier in a mouse model of colitis. Am J Physiol Gastrointest Liver Physiol. 2011;300(2):G327-33. doi: 10.1152/ajpgi.00422.2010 21109593 PMC3302190

[100] Johansson MEV, Jakobsson HE, Holmén-Larsson J, Schütte A, Ermund A, Rodríguez-Piñeiro AM, et al. Normalization of host intestinal mucus layers requires long-term microbial colonization. Cell Host Microbe. 2015;18(5):582–92. doi: 10.1016/j.chom.2015.10.007 26526499 PMC4648652

[101] Bergstrom KSB, Kissoon-Singh V, Gibson DL, Ma C, Montero M, Sham HP, et al. Muc2 protects against lethal infectious colitis by disassociating pathogenic and commensal bacteria from the colonic mucosa. PLoS Pathog. 2010;6(5):e1000902. doi: 10.1371/journal.ppat.1000902 20485566 PMC2869315

[102] Zarepour M, Bhullar K, Montero M, Ma C, Huang T, Velcich A, et al. The mucin Muc2 limits pathogen burdens and epithelial barrier dysfunction during *Salmonella enterica* serovar *Typhimurium colitis*. Infect Immun. 2013;81(10):3672–83. doi: 10.1128/IAI.00854-13 23876803 PMC3811786

[103] Zhang T, Sasabe J, Hullahalli K, Sit B, Waldor MK. Increased Listeria monocytogenes dissemination and altered population dynamics in Muc2-deficient mice. Infect Immun. 2021;89(4):e00667-20. doi: 10.1128/IAI.00667-20 33431704 PMC8090952

[104] Byndloss MX, Olsan EE, Rivera-Chávez F, Tiffany CR, Cevallos SA, Lokken KL, et al. Microbiota-activated PPAR-γ signaling inhibits dysbiotic Enterobacteriaceae expansion. Science. 2017;357(6351):570–5. doi: 10.1126/science.aam9949 28798125 PMC5642957

[105] Kelly CJ, Zheng L, Campbell EL, Saeedi B, Scholz CC, Bayless AJ, et al. Crosstalk between microbiota-derived short-chain fatty acids and intestinal epithelial HIF augments tissue barrier function. Cell Host Microbe. 2015;17(5):662–71. doi: 10.1016/j.chom.2015.03.005 25865369 PMC4433427

[106] Rivera-Chávez F, Zhang LF, Faber F, Lopez CA, Byndloss MX, Olsan EE, et al. Depletion of butyrate-producing Clostridia from the gut microbiota drives an aerobic luminal expansion of Salmonella. Cell Host Microbe. 2016;19(4):443–54. doi: 10.1016/j.chom.2016.03.004 27078066 PMC4832419

[107] Litvak Y, Byndloss MX, Tsolis RM, Bäumler AJ. Dysbiotic Proteobacteria expansion: a microbial signature of epithelial dysfunction. Curr Opin Microbiol. 2017;39:1–6. doi: 10.1016/j.mib.2017.07.003 28783509

[108] Marteyn B, West NP, Browning DF, Cole JA, Shaw JG, Palm F, et al. Modulation of *Shigella* virulence in response to available oxygen *in vivo*. Nature. 2010;465(7296):355–8. doi: 10.1038/nature08970 20436458 PMC3750455

[109] Lopez CA, Miller BM, Rivera-Chávez F, Velazquez EM, Byndloss MX, Chávez-Arroyo A, et al. Virulence factors enhance *Citrobacter rodentium* expansion through aerobic respiration. Science. 2016;353(6305):1249–53. doi: 10.1126/science.aag3042 27634526 PMC5127919

[110] Litvak Y, Mon KKZ, Nguyen H, Chanthavixay G, Liou M, Velazquez EM, et al. Commensal *Enterobacteriaceae* protect against *Salmonella* colonization through oxygen competition. Cell Host Microbe. 2019;25(1):128-139.e5. doi: 10.1016/j.chom.2018.12.003 30629913 PMC12036633

[111] Winter SE, Winter MG, Xavier MN, Thiennimitr P, Poon V, Keestra AM, et al. Host-derived nitrate boosts growth of *E. coli* in the inflamed gut. Science. 2013;339(6120):708–11. doi: 10.1126/science.1232467 23393266 PMC4004111

[112] Spees AM, Wangdi T, Lopez CA, Kingsbury DD, Xavier MN, Winter SE, et al. Streptomycin-induced inflammation enhances *Escherichia coli* gut colonization through nitrate respiration. mBio. 2013;4(4):e00430-13. doi: 10.1128/mBio.00430-13 23820397 PMC3705454

[113] Herp S, Brugiroux S, Garzetti D, Ring D, Jochum LM, Beutler M, et al. Mucispirillum schaedleri antagonizes *Salmonella* virulence to protect mice against colitis. Cell Host Microbe. 2019;25(5):681-694.e8. doi: 10.1016/j.chom.2019.03.004 31006637

[114] Vaishnava S, Yamamoto M, Severson KM, Ruhn KA, Yu X, Koren O, et al. The antibacterial lectin RegIIIgamma promotes the spatial segregation of microbiota and host in the intestine. Science. 2011;334(6053):255–8. doi: 10.1126/science.1209791 21998396 PMC3321924

[115] Cash HL, Whitham CV, Behrendt CL, Hooper LV. Symbiotic bacteria direct expression of an intestinal bactericidal lectin. Science. 2006;313(5790):1126–30. doi: 10.1126/science.1127119 16931762 PMC2716667

[116] Brandl K, Plitas G, Mihu CN, Ubeda C, Jia T, Fleisher M, et al. Vancomycin-resistant enterococci exploit antibiotic-induced innate immune deficits. Nature. 2008;455(7214):804–7. doi: 10.1038/nature07250 18724361 PMC2663337

[117] Abt MC, Buffie CG, Sušac B, Becattini S, Carter RA, Leiner I, et al. TLR-7 activation enhances IL-22-mediated colonization resistance against vancomycin-resistant enterococcus. Sci Transl Med. 2016;8(327):327ra25. doi: 10.1126/scitranslmed.aad6663 26912904 PMC4991618

[118] Waldschmitt N, Kitamoto S, Secher T, Zacharioudaki V, Boulard O, Floquet E, et al. The regenerating family member 3 β instigates IL-17A-mediated neutrophil recruitment downstream of NOD1/2 signalling for controlling colonisation resistance independently of microbiota community structure. Gut. 2019;68(7):1190–9. doi: 10.1136/gutjnl-2018-316757 30279238

[119] Mayneris-Perxachs J, Moreno-Navarrete JM, Fernández-Real JM. The role of iron in host-microbiota crosstalk and its effects on systemic glucose metabolism. Nat Rev Endocrinol. 2022;18(11):683–98. doi: 10.1038/s41574-022-00721-3 35986176

[120] Singh V, Yeoh BS, Chassaing B, Zhang B, Saha P, Xiao X, et al. Microbiota-inducible innate immune siderophore binding protein lipocalin 2 is critical for intestinal homeostasis. Cell Mol Gastroenterol Hepatol. 2016;2(4):482-498.e6. doi: 10.1016/j.jcmgh.2016.03.007 27458605 PMC4957954

[121] Klüber P, Meurer SK, Lambertz J, Schwarz R, Zechel-Gran S, Braunschweig T, et al. Depletion of lipocalin 2 (LCN2) in mice leads to dysbiosis and persistent colonization with segmented filamentous bacteria. Int J Mol Sci. 2021;22(23):13156. doi: 10.3390/ijms222313156 34884961 PMC8658549

[122] Zygiel EM, Nolan EM. Transition metal sequestration by the host-defense protein calprotectin. Annu Rev Biochem. 2018;87:621–43. doi: 10.1146/annurev-biochem-062917-012312 29925260 PMC6066180

[123] Ivanov II, Atarashi K, Manel N, Brodie EL, Shima T, Karaoz U, et al. Induction of intestinal Th17 cells by segmented filamentous bacteria. Cell. 2009;139(3):485–98. doi: 10.1016/j.cell.2009.09.033 19836068 PMC2796826

[124] Kernbauer E, Ding Y, Cadwell K. An enteric virus can replace the beneficial function of commensal bacteria. Nature. 2014;516(7529):94–8. doi: 10.1038/nature13960 25409145 PMC4257755

[125] Neil JA, Matsuzawa-Ishimoto Y, Kernbauer-Hölzl E, Schuster SL, Sota S, Venzon M, et al. IFN-I and IL-22 mediate protective effects of intestinal viral infection. Nat Microbiol. 2019;4(10):1737–49. doi: 10.1038/s41564-019-0470-1 31182797 PMC6871771

[126] Macpherson AJ, Gatto D, Sainsbury E, Harriman GR, Hengartner H, Zinkernagel RM. A primitive T cell-independent mechanism of intestinal mucosal IgA responses to commensal bacteria. Science. 2000;288(5474):2222–6. doi: 10.1126/science.288.5474.2222 10864873

[127] Bunker JJ, Flynn TM, Koval JC, Shaw DG, Meisel M, McDonald BD, et al. Innate and adaptive humoral responses coat distinct commensal bacteria with immunoglobulin A. Immunity. 2015;43(3):541–53. doi: 10.1016/j.immuni.2015.08.007 26320660 PMC4575282

[128] Bunker JJ, Erickson SA, Flynn TM, Henry C, Koval JC, Meisel M, et al. Natural polyreactive IgA antibodies coat the intestinal microbiota. Science. 2017;358(6361):eaan6619. doi: 10.1126/science.aan6619 28971969 PMC5790183

[129] Lopez CA, Winter SE, Rivera-Chávez F, Xavier MN, Poon V, Nuccio S-P, et al. Phage-mediated acquisition of a type III secreted effector protein boosts growth of *Salmonella* by nitrate respiration. mBio. 2012;3(3):e00143-12. doi: 10.1128/mBio.00143-12 22691391 PMC3374392

[130] Faber F, Tran L, Byndloss MX, Lopez CA, Velazquez EM, Kerrinnes T, et al. Host-mediated sugar oxidation promotes post-antibiotic pathogen expansion. Nature. 2016;534(7609):697–9. doi: 10.1038/nature18597 27309805 PMC4939260

[131] Zhao W, Caro F, Robins W, Mekalanos JJ. Antagonism toward the intestinal microbiota and its effect on *Vibrio cholerae* virulence. Science. 2018;359(6372):210–3. doi: 10.1126/science.aap8775 29326272 PMC8010019

[132] Sana TG, Flaugnatti N, Lugo KA, Lam LH, Jacobson A, Baylot V, et al. *Salmonella Typhimurium* utilizes a T6SS-mediated antibacterial weapon to establish in the host gut. Proc Natl Acad Sci U S A. 2016;113(34):E5044-51. doi: 10.1073/pnas.1608858113 27503894 PMC5003274

[133] Anderson MC, Vonaesch P, Saffarian A, Marteyn BS, Sansonetti PJ. *Shigella sonnei* encodes a functional T6SS used for interbacterial competition and niche occupancy. Cell Host Microbe. 2017;21(6):769-776.e3. doi: 10.1016/j.chom.2017.05.004 28618272

[134] Serapio-Palacios A, Woodward SE, Vogt SL, Deng W, Creus-Cuadros A, Huus KE, et al. Type VI secretion systems of pathogenic and commensal bacteria mediate niche occupancy in the gut. Cell Rep. 2022;39(4):110731. doi: 10.1016/j.celrep.2022.110731 35476983

[135] Guo R, Fang X, Shang K, Wen J, Ding K. Induction of ferroptosis: a new strategy for the control of bacterial infections. Microbiol Res. 2024;284:127728. doi: 10.1016/j.micres.2024.127728 38643523

[136] Tailford LE, Crost EH, Kavanaugh D, Juge N. Mucin glycan foraging in the human gut microbiome. Front Genet. 2015;6:81. doi: 10.3389/fgene.2015.00081 25852737 PMC4365749

[137] Derrien M, van Passel MW, van de Bovenkamp JH, Schipper RG, de Vos WM, Dekker J. Mucin-bacterial interactions in the human oral cavity and digestive tract. Gut Microbes. 2010;1(4):254–68. doi: 10.4161/gmic.1.4.12778 21327032 PMC3023607

[138] Yamauchi J, Kawai Y, Yamada M, Uchikawa R, Tegoshi T, Arizono N. Altered expression of goblet cell- and mucin glycosylation-related genes in the intestinal epithelium during infection with the nematode *Nippostrongylus brasiliensis* in rat. APMIS. 2006;114(4):270–8. doi: 10.1111/j.1600-0463.2006.apm_353.x 16689826

[139] Soga K, Yamauchi J, Kawai Y, Yamada M, Uchikawa R, Tegoshi T, et al. Alteration of the expression profiles of acidic mucin, sialytransferase, and sulfotransferases in the intestinal epithelium of rats infected with the nematode *Nippostrongylus brasiliensis*. Parasitol Res. 2008;103(6):1427–34. doi: 10.1007/s00436-008-1152-8 18716796

[140] Hasnain SZ, Dawson PA, Lourie R, Hutson P, Tong H, Grencis RK, et al. Immune-driven alterations in mucin sulphation is an important mediator of *Trichuris muris* helminth expulsion. PLoS Pathog. 2017;13(2):e1006218. doi: 10.1371/journal.ppat.1006218 28192541 PMC5325613

[141] Ramanan D, Bowcutt R, Lee SC, Tang MS, Kurtz ZD, Ding Y, et al. Helminth infection promotes colonization resistance via type 2 immunity. Science. 2016;352(6285):608–12. doi: 10.1126/science.aaf3229 27080105 PMC4905769

[142] Hu Z, Zhang C, Sifuentes-Dominguez L, Zarek CM, Propheter DC, Kuang Z, et al. Small proline-rich protein 2A is a gut bactericidal protein deployed during helminth infection. Science. 2021;374(6568):eabe6723. doi: 10.1126/science.abe6723 34735226 PMC8977106

[143] Propheter DC, Chara AL, Harris TA, Ruhn KA, Hooper LV. Resistin-like molecule β is a bactericidal protein that promotes spatial segregation of the microbiota and the colonic epithelium. Proc Natl Acad Sci U S A. 2017;114(42):11027–33. doi: 10.1073/pnas.1711395114 28973871 PMC5651776

[144] Venzon M, Das R, Luciano DJ, Burnett J, Park HS, Devlin JC, et al. Microbial byproducts determine reproductive fitness of free-living and parasitic nematodes. Cell Host Microbe. 2022;30(6):786-797.e8. doi: 10.1016/j.chom.2022.03.015 35413267 PMC9187612

[145] Roager HM, Hansen LBS, Bahl MI, Frandsen HL, Carvalho V, Gøbel RJ, et al. Colonic transit time is related to bacterial metabolism and mucosal turnover in the gut. Nat Microbiol. 2016;1(9):16093. doi: 10.1038/nmicrobiol.2016.93 27562254

[146] White EC, Houlden A, Bancroft AJ, Hayes KS, Goldrick M, Grencis RK, et al. Manipulation of host and parasite microbiotas: survival strategies during chronic nematode infection. Sci Adv. 2018;4(3):eaap7399. doi: 10.1126/sciadv.aap7399 29546242 PMC5851687

[147] Yang CM, Ferket PR, Hong QH, Zhou J, Cao GT, Zhou L, et al. Effect of chito-oligosaccharide on growth performance, intestinal barrier function, intestinal morphology and cecal microflora in weaned pigs. J Anim Sci. 2012;90(8):2671–6. doi: 10.2527/jas.2011-4699 22785166

[148] Guan G, Wang H, Chen S, Liu G, Xiong X, Tan B, et al. Dietary chitosan supplementation increases microbial diversity and attenuates the severity of *Citrobacter rodentium* infection in mice. Mediators Inflamm. 2016;2016:9236196. doi: 10.1155/2016/9236196 27761062 PMC5059534

[149] Tielens AGM, van Grinsven KWA, Henze K, van Hellemond JJ, Martin W. Acetate formation in the energy metabolism of parasitic helminths and protists. Int J Parasitol. 2010;40(4):387–97. doi: 10.1016/j.ijpara.2009.12.006 20085767

[150] Gause WC, Maizels RM. Macrobiota—helminths as active participants and partners of the microbiota in host intestinal homeostasis. Curr Opin Microbiol. 2016;32:14–8. doi: 10.1016/j.mib.2016.04.004 27116368 PMC4983462

[151] Moyat M, Lebon L, Perdijk O, Wickramasinghe LC, Zaiss MM, Mosconi I, et al. Microbial regulation of intestinal motility provides resistance against helminth infection. Mucosal Immunol. 2022;15(6):1283–95. doi: 10.1038/s41385-022-00498-8 35288644 PMC9705251

[152] Harris NL, Loke P. Recent advances in type-2-cell-mediated immunity: insights from helminth infection. Immunity. 2017;47(6):1024–36. doi: 10.1016/j.immuni.2017.11.015 29262347

